# Combined Endovascular and Surgical Treatment of Chronic Carotid Artery Occlusion: Hybrid Operation

**DOI:** 10.1155/2020/6622502

**Published:** 2020-11-28

**Authors:** Long Yan, Zhe Wang, Zhanchuan Liu, Haoyuan Yin, Xuan Chen

**Affiliations:** ^1^Department of Interventional Neuroradiology, Beijing Tiantan Hospital, Capital Medical University, Beijing 100000, China; ^2^Department of Gerontology, The First Hospital of Jilin University, Changchun, Jilin 130021, China; ^3^Department of Neurosurgery, The Second Hospital of Jilin University, Changchun, Jilin 130041, China; ^4^Department of Neurosurgery, The First Hospital of Jilin University, Changchun, Jilin 130021, China

## Abstract

**Objectives:**

The optimal treatment choice of chronic carotid artery occlusion (CAO) remains inconclusive. This study was aimed at exploring the safety and effectiveness of hybrid surgery in the treatment of CAO and at determining predictors for successful recanalization.

**Methods:**

In this study, we enrolled 37 patients with CAO who underwent hybrid surgical treatment during the period 2016–2018. We extracted and analyzed patients' demographic data, disease characteristics, surgical success rates, perioperative complications, and prognosis.

**Results:**

A total of 37 patients with symptomatic CAO underwent hybrid surgical treatment. Thirty cases (81.1%) were successfully recanalized, while seven were not. Blood reflux after carotid endarterectomy occurred in 18 patients (60%) of the success group and 1 (14.3%) of the failure group (OR, 9.0; 95% CI, 0.95-54.5; *P* = 0.042). The rate of distal ICA reconstruction below the clinoid segment was 20 (66.7%) in the success group and 1 (14.3%) in the failure group (OR, 12.0; 95% CI, 1.3-113.7; *P* = 0.029). In patients with successful recanalization, no ischemic events occurred after surgery and during follow-up, but restenosis of >50% was found in one case. In the failure group, two patients experienced recurrent ischemic events during follow-up. Perfusion imaging in successful recanalization cases is significantly improved, preoperative I/C ratio was 1.44 (IQR 1.27-1.55), and postoperative 1.12 (IQR 1.05-1.23). National Institutes of Health Stroke Scale (NIHSS) score of successful recanalization cases was 5.35 (2.26) before surgery and 2.03 (1.40) at 6 months (*P* < 0.01).

**Conclusion:**

Hybrid surgery might be a safe and effective way to treat CAO. Distal internal carotid artery reconstruction to below the clinoid segment and blood reflux after carotid endarterectomy are predictors of successful recanalization.

## 1. Introduction

Chronic carotid artery occlusion (CAO) is an uncommon but important cause of ischemic stroke. The natural course of CAO varies greatly, and it can manifest as stroke, transient ischemic attack (TIA), or no symptoms at all. Territories of which blood supply is occluded rely on compensating collateral flow, but for some patients, these are not sufficient. Among patients with hemodynamic disorders, those with CAO have significantly higher rates of recurrent stroke and mortality [1]. Treatment of CAO is still controversial. Alternative treatments are as follows: conservative treatment including antiplatelets and anticoagulants, extracranial-intracranial (EC-IC) bypass, carotid endarterectomy (CEA), and endovascular recanalization.

Hybrid surgery refers to the combination of endovascular recanalization and CEA. Reaching the distal true lumen through the occluded segment is the key to successful recanalization in CAO. Hybrid surgery combines their advantages and overcomes their respective difficulties. CEA removes plaque, allowing the microcatheter and microguidewire to easily pass through to the distal true lumen. Some studies have reported the application of hybrid surgery in CAO [2–4], but the predictors of successful recanalization have not been fully discussed. The purposes of the study are to explore the effectiveness and safety of hybrid surgery for CAO and the predictors of successful recanalization.

## 2. Materials and Methods

### 2.1. Patient Selection

This retrospective study was approved by the local institutional review board. We included symptomatic CAO patients who were hospitalized in the Department of Neurosurgery of the First Hospital of Jilin University (Changchun, China) from January 2016 to December 2018. CAO was defined as a duration of ≥4 weeks between diagnosis and treatment. Inclusion criteria were as follows: (1) 18–80 years old, (2) complete carotid artery occlusion as confirmed by digital-subtraction angiography (DSA), (3) TIA or stroke occurring in the occluded blood vessel supply area over the past 12 months, (4) computed tomography (CT) or magnetic resonance (MR) perfusion imaging confirming hypoperfusion on the occluded side, (5) CT or MR excluding extensive cerebral infarction, and (6) cases with complete clinical and imaging data to permit follow-up. Exclusion criteria were as follows: (1) severe diseases of the heart, liver, kidney, lung, or other vital organ, with estimated survival time < 3 months; (2) inability to tolerate general anesthesia for surgery; (3) allergy to contrast agents; (4) contraindication of, or resistance to, aspirin and/or clopidogrel; (5) other cerebrovascular diseases (intracranial aneurysms and cerebrovascular malformations) causing stroke; and (6) severe neurological dysfunction and impaired consciousness.

### 2.2. Data Collection

**Figure 1:**
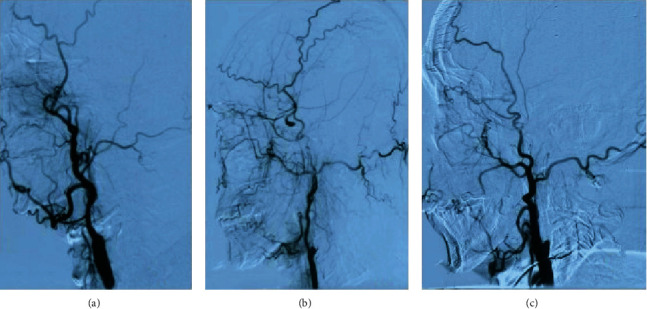
Stump condition: (a) no stump; (b) blunt stump; (c) tapered stump.

Baseline information included sex, age, diabetes, hypertension, smoking, drinking, and hyperlipidemia. NIHSS score on admission and duration from the last neurologic event to surgery was also collected. All imaging data are assessed individually by two neurosurgeons. All patients underwent CT perfusion imaging or MR perfusion imaging before and one week after surgery. Mean transit times (MTTs) of ipsilateral- (side of the occluded ICA-) to-contralateral (I/C) were calculated. The morphological description included the following: stump morphology: (1) blunt stump or no stump and (2) tapered stump (Figure 1). Compensatory vessels included the following: anterior communicating artery, posterior communicating artery, ipsilateral ophthalmic artery, and branches of other arteries. Reflux sites were categorized as follows: (1) below the clinoid segment (cavernous segment, petrous segment, and below) and (2) clinoid segment and above (clinoid segment, ophthalmic segment, or communicating segment). Surgical complications included hyperperfusion, ischemic events, hemorrhage events, carotid cavernous fistula, dissection, and so on.

### 2.3. Hybrid Surgical Procedure

**Figure 2:**
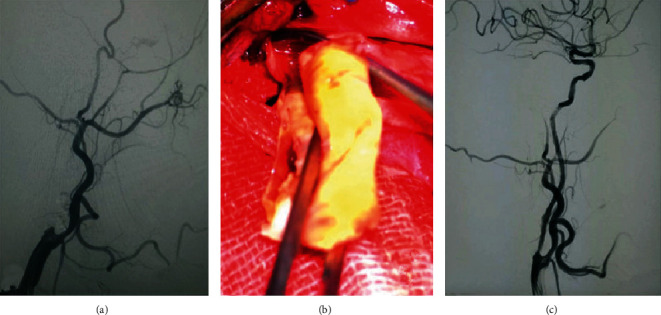
(a) Preoperative chronic carotid artery occlusion. (b) Remove carotid plaque. (c) Carotid artery recanalization after hybrid surgery.

After general anesthesia, patients were placed in the supine position with their heads tilted to the opposite side from the lesion. A straight incision was made at the front edge of the sternocleidomastoid muscle on the affected side to cut through the skin and platysma muscle after routine disinfection. We separated the skin and muscle along the front edge of the sternocleidomastoid muscle to the ICA sheath so that we could expose and cut the fascia of the ICA in order to lift that artery. The superior thyroid artery, external carotid artery, and distal end of the ICA were clamped in turn. Afterwards, we cut the common carotid artery (CCA) along the long axis to the distal end of the ICA. After stripping off the intima of the ICA containing the plaque and thrombus and then flushing and thoroughly stripping the lumen, we observed the distal end for blood reflux. After stitching up the vessel wall, an 8F catheter sheath was inserted into the right femoral artery to support the catheter to the bifurcation of the CCA. Via angiography, we observed the recanalization status of the distal blood vessels. If it remained occluded, we passed a microcatheter and microguidewire through to the occluded lumen in order to determine stenotic and occluded sites. Then, balloon-angioplasty was used to expand the blood vessel at the site of occlusion and stenosis, followed by insertion and release of the appropriate stent. A postsurgery blood flow reaching Thrombolysis in Cerebral Infarction (TICI) grade IIb or higher is defined as successful recanalizaiton (Figure 2).

### 2.4. Follow-Up

All patients were recommended for follow-up. Clinical follow-up content is as follows: all cases are recommended for outpatient or telephone follow-up and we record the patients' NIHSS score and record hemorrhage and ischemic events. Image follow-up content is as follows: color Doppler ultrasound at 1 month, CTA at 3 months, and DSA at 6 months to record whether the recanalized blood vessel is unobstructed, whether there is reocclusion or restenosis, and whether there are other vascular injuries, such as carotid artery dissection and pseudo aneurysm, and carotid cavernous fistula.

### 2.5. Statistical Analysis

We performed statistical analysis using SPSS software version 20.0 (IBM Corp., Armonk, New York, US). Normal distribution measurement data were expressed as means and standard deviations (SD), while skew distribution data were expressed as the median and interquartile range (IQR). Counting data were expressed in frequencies and percentages. We compared data between each group using Student's *t* test (normal distribution) or the Mann-Whitney *U* test (skewed distribution), the chi-square test, or Fisher's exact test according to the situation. *P* < 0.05 was considered a statistically significant difference.

## 3. Results and Discussion

### 3.1. Results

**Figure 3:**
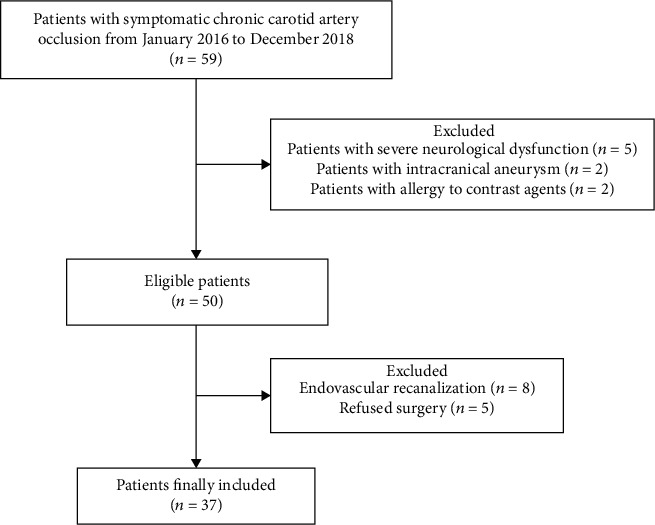
Selection of patients.

Of the 59 patients with symptomatic CAO who were admitted during the study period, a total of 37 patients were included in the study (Figure 3). Thirty cases (81.1%) were successfully recanalized and seven were not. Age, male sex, hypertension, diabetes, hyperlipidemia, smoking, and drinking did not differ significantly between the success and failure groups (*P* > 0.05). Male patients accounted for 89.1% of total cases, a significantly higher proportion than that of female patients. The majority of patients smoked (70.3%). Duration from last onset to surgery was 15.0 (IQR 14.0-33.75) days in cases of the success group and 60.0 (IQR 30.0-120.0) in those of the failure group (*P* = 0.007); therefore, disease last onset was longer in the failure group than in the success group (Table 1).

The lesion was on the left side in 18 cases and on the right side in 19. The occlusion was located in the CCA in 4 cases (10.8%), in the beginning of ICA in 27 cases (73.0%), and in other sites in 6 cases (16.2%). Most of the occlusions were located at the beginning of the ICA. Twenty cases had a tapered stump, and the other 17 cases had a blunt or no stump at all. After CEA, 19 cases showed blood reflux, with 18 successful recanalizations and 1 failure. The other 18 cases showed no blood reflux, with 12 successful recanalizations and 6 failures. (*P* = 0.042). In 16 cases of distal ICA reconstruction at the clinoid segment and above, 10 cases were successfully recanalized and 6 cases were not recanalized; 21 cases below the clinoid segment were successfully recanalized in 20 cases and 1 case was not recanalized (*P* = 0.029) (Table 2).

There were 2 cases of intracranial hemorrhage, 1 case died of sudden breathing, cardiac arrest, and cerebral hemorrhage on the second day after operation; 1 case died of intracranial hemorrhage caused by rupture of the blood vessel during operation. Two cases showed hyperperfusion, which was relieved after medication. There were no ischemic events in the success group after surgery or during follow-up, but restenosis of >50% was found in one case. Of the seven patients with failed recanalization, two had recurrent ischemic events during follow-up (Table 3).

Preoperative I/C ratio in success group cases was 1.44 (IQR 1.27-1.55); 1 week after surgery, the I/C ratio in these cases was 1.12 (IQR 1.05-1.23), *P* < 0.01. Cerebral perfusion in success group cases was significantly improved. Preoperative NIHSS score in success group cases was 5.35 (2.26); at 6-month follow-up, NIHSS score in these cases was 2.03 (1.40), *P* < 0.01. Neurological function was significantly improved in postoperative patients (Table 4).

### 3.2. Discussion

Even with intensive drug treatment, the CAO annual stroke recurrence rate is 6% to 20% [1, 5]. It is necessary to establish an alternative treatment with an acceptable safety and efficacy profile. In this study, the recanalization rate of hybrid surgery was 81.1%, with a relatively low risk of complications. We found that distal ICA reconstruction to below the clinoid segment and blood reflux after carotid endarterectomy are predictors of successful recanalization.

Various treatment methods for CAO have developed gradually since the 1960s, including bypass surgery, CEA, and intravascular recanalization under interventional radiology. EC-IC bypass is not recommended duo to failing to reduce the risk of recurrent ipsilateral ischemic stroke within 2 years compared with medical therapy [6]. CEA, due to anatomical limitations, is applicable only for occlusion at the origin of the internal carotid artery (ICA). For long-segment occlusions that extend to the intracranial segment, pure CEA presents challenges to successful recanalization [7]. With the development of neurointerventional technology and materials, endovascular recanalization has been more frequently explored, and more studies have reported the improved possibility of successful endovascular recanalization [8–10]. In one study enrolling 138 patients who underwent endovascular recanalization surgeries, 85 of them (61.6%) were technically successful [11]. However, 6 patients experienced recurrent stroke, death, or intracranial hemorrhage, and 11 (8.0%) had intracranial carotid cavernous fistulae. Thus, the recanalization success rate is still unsatisfactory and the complication rate still high [12].

CEA for the treatment of CAO is limited to occlusion location at places that can be exposed during operation while no long-segment thrombosis exists in the distal blood vessels, which restrict its scope of application. As to endovascular treatment, it was first reported in 2013 for CAO patients [2]; all three patients were successfully recanalized. The cerebral-perfusion imaging showed significant improvement, and no ischemic events occurred during the 6 months of follow-up. For the past few years, hybrid surgery, combining the CEA and endovascular treatment, has gradually developed to treat CAO. The occlusion site is more likely to locate at the beginning of the ICA for CAO. CEA removes the plaque at the origin ICA, allowing the microcatheter and microguidewire to easily pass through to the distal true lumen. This paves the way for endovascular interventional recanalization, which in turn treats the distal carotid artery lesion using balloons and stents. Theoretically, combing the advantages of the two surgical modalities may improve the success rate. Liu et al. reported [13] that of 21 patients who received hybrid surgical treatment, 15 (71.4%) were successfully recanalized without causing carotid dissections, intracranial hemorrhages, or new neurological deficits. In the current study, we enrolled a total of 37 patients and 30 (81.1%) were successfully recanalized. Therefore, it showed that hybrid surgery may be an effective surgical method for CAO.

Many studies reported the successful recanalization of CAO with hybrid surgery [2–4, 13], but few reported the predictors for successful recanalization. In this study, distal ICA reconstruction to below the clinoid segment and blood reflux after carotid CEA are predictors of successful recanalization. Both of these predictors indicate a shorter occlusion length. Hybrid surgery makes it possible to reach the distal true lumen through the occluded segment. The occlusion length may be another key factor of successful recanalization. We need more cases and more advanced inspection methods such as high resolution magnetic resonance imaging to verify.

The goal of treatment is to restore blood supply, improve hypoperfusion, and reduce the incidence of recurrent stroke. Studies have shown that hemodynamic damage in patients with CAO is significantly correlated with the incidence of stroke [14, 15]. CT/MR perfusion imaging has in fact been widely used in clinical practice for pre-and postevaluation of CAO cases. A study comparing CT perfusion imaging with positron emission tomography (PET) perfusion imaging in CAO patients showed that MTT parameters of CT perfusion imaging were significantly correlated with oxygen extraction fraction (OEF) as measured by PET [16]. Mukherjee et al. reported [17] that MR perfusion imaging in patients with CAO was linearly correlated with cerebral blood flow (CBF) as measured by PET. Due to the large differences in absolute-value measurement between different modalities, semiquantitative comparisons with the ipsilateral side of the lesion are mostly used at present, and their accuracy has been recognized [18]. All patients in this study underwent CT or MR perfusion before surgery and again 1 week after surgery. Successful cases had a preoperative I/C ratio of 1.44 (IQR 1.27-1.55) and a postoperative ratio I/C of 1.12 (IQR 1.05-1.23), which showed that perfusion was significantly improved before and after surgery.

There were some limitations to this study. First, it was a retrospective study and it is inevitable that the collection of relevant data will be biased; second, the number of cases in this study was small and could not be stratified in more detail based on the level of distal ICA reconstitution. Due to the small sample size, statistical methods used in this study were Student's *t* test (normal distribution) or Mann-Whitney *U* test (skewed distribution), the chi-square test, or Fisher's exact test; univariate and multivariate analyses could not be performed.

## 4. Conclusions

Hybrid surgery may be a safe and effective method of treating CAO, with a higher success rate and fewer complications than traditional methods. Distal ICA reconstruction to below the clinoid segment and blood reflux after carotid endarterectomy are predictors of successful recanalization.

## Figures and Tables

**Table 1:** Baseline characteristics of patients.

	Total (*n* = 37)	Success group (*n* = 30)	Failure group (*n* = 7)	*P* value
Age, mean (SD)	62.1 (6.4)	62.9 (6.4)	58.9 (6.1)	0.140
Male, *n* (%)	33 (89.1%)	27 (90.0%)	6 (85.7%)	1.000
Hypertension, *n* (%)	24 (64.9%)	21 (70.0%)	3 (42.9%)	0.213
Diabetes, *n* (%)	20 (54.1%)	16 (53.3%)	4 (57.1%)	1.000
Hyperlipidemia, *n* (%)	19 (51.4%)	15 (50.0%)	4 (57.1%)	1.000
Smoking, *n* (%)	26 (70.3%)	21 (70.0%)	5 (71.4%)	1.000
Drinking, *n* (%)	16 (43.2%)	14 (46.7%)	2 (28.6%)	0.674
Duration from last onset to surgery, median (IQR)	30.0 (14.5-47.5)	15.0 (14.0-33.75)	60.0 (30.0-120.0)	0.007

**Table 2:** Lesion characteristics of patients.

	Success group (*n* = 30)	Failure group (*n* = 7)	OR (95% CI)	*P* value
Left sided, *n* (%)	16 (53.3%)	2 (28.6%)	2.9 (0.5-17.1)	0.405
Occlusion site			—	0.085
CCA, *n* (%)	4 (13.3%)	0 (0.0%)		
Beginning of ICA, *n* (%)	23 (76.7%)	4 (57.1%)		
Other sites, *n* (%)	3 (10.0%)	3 (42.9%)		
Stump condition			0.9 (0.2-4.5)	1.000
Tapered, *n* (%)	16 (53.3%)	4 (57.1%)		
Blunt or no stump, *n* (%)	14 (46.7%)	3 (42.9%)		
Blood reflux after CEA, *n* (%)	18 (60.0%)	1 (14.3%)	9.0 (0.95-54.5)	0.042
Level of distal ICA reconstruction			12.0 (1.3-113.7)	0.029
Clinoid segment and beyond, *n* (%)	10 (33.3%)	6 (85.7%)		
Below clinoid segment, *n* (%)	20 (66.7%)	1 (14.3%)		

CCA: common carotid artery; ICA: internal carotid artery; CEA: carotid endarterectomy.

**Table 3:** Postoperative complications and follow up.

	Success group (*n* = 30)	Failure group (*n* = 7)
Hyperperfusion, *n* (%)	2 (6.7%)	0 (0.0%)
Ischemic stroke, *n* (%)	0 (0.0%)	2 (28.6%)
Hemorrhage, *n* (%)	1 (3.3%)	1 (14.3%)
Death, *n* (%)	1 (3.3%)	1 (14.3%)

**Table 4:** Imaging improvement and functional improvement.

	Success group		*P*
	Preoperative	Postoperative	
I/C, median (IQR)	1.44 (1.27-1.55)	1.12 (1.05-1.23)	<0.01
NIHSS, mean (SD)	5.35 (2.26)	2.03 (1.40)	<0.01

I/C: mean transit times ipsilateral-to-contralateral; NIHSS: National Institutes of Health Stroke Scale.

## Data Availability

The data that support the findings of this study are available from the corresponding author upon reasonable request.

## Supplementary Materials

Supplementary Materials See table in the Supplementary Material for comprehensive data analysis.
