# Supplementary material for: Combined Endovascular and Surgical Treatment of Chronic Carotid Artery Occlusion: Hybrid Operation
Source: Biomed Res Int. 2020 Nov 28;2020:6622502. doi: 10.1155/2020/6622502 (PMC7723474; doi:10.1155/2020/6622502)
Supplement: Supplementary Materials — See table in the Supplementary Material for comprehensive data analysis. [file 6622502.f1.pdf]

	Sex	Age	lypertentior	Diabetes	Hyperlipidemia	Smoking	Drinking	Duration
1	M	50	Y	Y	Y	Y	N	50
2	M	59	Y	N	Y	N	N	21
3	M	56	Y	Y	N	Y	Y	45
4	M	60	N	Y	N	Y	Y	60
5	M	69	N	N	Y	Y	Y	7
6	M	62	Y	N	Y	Y	N	120
7	M	47	Y	Y	N	Y	Y	17
8	M	57	N	N	N	Y	Y	30
9	M	62	N	Y	N	Y	Y	30
10	M	67	Y	Y	Y	Y	N	15
11	M	61	Y	Y	Y	Y	Y	30
12	M	77	Y	N	N	N	N	60
13	M	71	Y	Y	Y	Y	Y	10
14	M	67	Y	N	N	Y	N	30
15	M	62	Y	Y	N	Y	Y	90
16	F	73	Y	Y	Y	N	N	30
17	M	66	Y	Y	Y	Y	Y	7
18	M	50	N	Y	Y	Y	N	90
19	M	52	N	Y	Y	N	N	30
20	M	66	N	N	Y	Y	Y	15
21	M	65	N	N	N	Y	Y	13
22	M	64	Y	N	Y	Y	N	240
23	M	65	Y	N	N	Y	N	15
24	M	59	N	Y	Y	Y	N	5
25	M	61	N	N	N	N	N	15
26	M	62	Y	N	Y	Y	Y	14
27	F	64	Y	N	Y	Y	N	120
28	M	70	N	Y	N	Y	N	14
29	F	60	N	Y	Y	Y	N	14
30	F	60	Y	N	N	N	N	15
31	M	65	Y	Y	N	Y	Y	15
32	M	65	Y	Y	N	N	N	30
33	M	58	Y	N	N	Y	Y	120
34	M	63	Y	N	Y	N	N	15
35	M	69	N	Y	Y	N	Y	10
36	M	56	Y	N	N	N	N	30
37	M	58	Y	Y	N	N	N	45

Left/Right	Stump condition	Blood reflux	Distal ICA reconstruction	Occlusion site
L	Blunt or no stump	N	Clinoid segment and beyond	ICA
L	Blunt or no stump	Y	Below clinoid segment	CCA
L	Tapered	N	Below clinoid segment	ICA
R	Tapered	N	Clinoid segment and beyond	Others
L	Tapered	Y	Below clinoid segment	ICA
R	Blunt or no stump	Y	Clinoid segment and beyond	ICA
L	Tapered	Y	Below clinoid segment	ICA
R	Tapered	N	Clinoid segment and beyond	Others
L	Blunt or no stump	Y	Below clinoid segment	ICA
R	Blunt or no stump	Y	Below clinoid segment	ICA
R	Tapered	N	Clinoid segment and beyond	ICA
L	Blunt or no stump	Y	Below clinoid segment	CCA
R	Blunt or no stump	Y	Below clinoid segment	ICA
R	Tapered	N	Clinoid segment and beyond	ICA
L	Tapered	N	Clinoid segment and beyond	ICA
L	Tapered	Y	Below clinoid segment	CCA
L	Tapered	N	Below clinoid segment	ICA
L	Blunt or no stump	N	Clinoid segment and beyond	ICA
R	Tapered	N	Clinoid segment and beyond	Others
R	Tapered	Y	Below clinoid segment	ICA
R	Blunt or no stump	N	Clinoid segment and beyond	ICA
R	Blunt or no stump	N	Clinoid segment and beyond	ICA
L	Blunt or no stump	Y	Below clinoid segment	ICA
L	Blunt or no stump	Y	Below clinoid segment	ICA
R	Tapered	N	Clinoid segment and beyond	ICA
L	Tapered	Y	Below clinoid segment	ICA
L	Tapered	Y	Below clinoid segment	ICA
L	Tapered	Y	Below clinoid segment	ICA
R	Blunt or no stump	Y	Below clinoid segment	ICA
R	Blunt or no stump	Y	Below clinoid segment	CCA
R	Tapered	N	Clinoid segment and beyond	Others
R	Blunt or no stump	N	Clinoid segment and beyond	ICA
L	Blunt or no stump	Y	Below clinoid segment	ICA
R	Blunt or no stump	N	Below clinoid segment	ICA
L	Tapered	Y	Below clinoid segment	ICA
R	Tapered	N	Clinoid segment and beyond	Others
R	Tapered	N	Clinoid segment and beyond	ICA

Success	Preoperative I/C	Postoperative I/C	Preoperative NHISS	Postoperative NHISS
Y	1.56	1.21	6	2
Y	1.37	1.03	5	1
Y	1.28	1.05	5	3
N	1.41	1.25	8	6
Y	1.78	1.15	10	5
Y	1.44	1.22	8	4
Y	1.76	1.32	5	2
N	1.45	1.38	5	4
Y	1.22	1.08	6	4
Y	1.11	1.02	3	2
Y	1.32	1.01	2	0
Y	1.23	1.06	6	3
Y	1.48	1.08	4	1
Y	1.37	1.09	3	1
Y	1.06	1	4	1
Y	1.1	1	2	0
Y	1.16	1.02	4	1
N	1.35	1.28	5	4
N	1.58	Dead	9	Dead
Y	1.49	Dead	6	Dead
Y	1.69	1.27	3	0
N	1.85	2.82	3	2
Y	1.41	1.1	3	0
Y	1.14	1.01	8	2
Y	1.39	1.05	6	2
Y	1.46	1.13	9	3
N	1.53	1.5	5	3
Y	1.5	1.21	7	4
Y	1.98	1.23	10	4
Y	1.41	1.12	2	0
Y	1.51	1.11	4	3
N	1.21	1.13	4	2
Y	2.36	1.51	5	2
Y	1.26	1.08	6	2
Y	1.53	1.14	6	2
Y	1.57	1.16	7	3
Y	1.46	1.08	6	2
